# Cognitive testing of the PHQ-9 for depression screening among pregnant and postpartum women in Kenya

**DOI:** 10.1186/s12888-020-2435-6

**Published:** 2020-01-29

**Authors:** Jennifer Velloza, John Njoroge, Kenneth Ngure, Nicholas Thuo, Catherine Kiptinness, Richard Momanyi, Snaidah Ayub, Stephen Gakuo, Nelly Mugo, Jane Simoni, Renee Heffron

**Affiliations:** 1 Department of Global Health, University of Washington, Seattle, USA; 2 Department of Epidemiology, University of Washington, Seattle, USA; 3 International Clinical Research Center, University of Washington, Box 359927, 325 Ninth Avenue, Seattle, WA 98104 USA; 4 Partners in Health and Research Development, Nairobi, Kenya; 5 Department of Community Health Jomo Kenyatta University of Agriculture and Technology, Nairobi, Kenya; 6 Center for Clinical Research, Kenya Medical Research Institute, Nairobi, Kenya; 7 Department of Psychology, University of Washington, Seattle, USA

**Keywords:** Depression, PHQ-9, Pregnancy, Kenya, Women

## Abstract

**Background:**

African women face high rates of depression, particularly during pregnancy or postpartum or after a recent HIV diagnosis. The Patient Health Questionnaire-9 (PHQ-9) depression screening tool has been quantitatively validated and extensively used to identify depression and link individuals to care. However, qualitative work is necessary to identify important opportunities to improve PHQ-9 question comprehension and performance among Kenyan women.

**Methods:**

We administered the Kiswahili or English PHQ-9 (based on preference) to 29 pregnant and postpartum women in Thika, Kenya. Following administration, we conducted cognitive interviews with a purposive sample of 20 women. We used analytic memos and data matrices to identify themes around scale acceptability, comprehension, and decision and response processes.

**Results:**

Most participants preferred to answer the PHQ-9 in Kiswahili (*N* = 15; 52%). Among the 20 interview participants, 12 (60%) had scores ≥5, indicating depressive symptoms. Overall, participants found the scale acceptable as an interviewer-administered tool. Participants reported few problems related to comprehension but had difficulty answering items not relevant to their lives (e.g., “watching television”) and double-barreled items (e.g., “poor appetite or overeating”). They were hesitant to endorse items related to “duties as a wife and mother” and suicidal ideation. Most participants had difficulty distinguishing between response options of “several days” and “more than half the days”.

**Conclusions:**

We detected several problems related to PHQ-9 comprehension, decision processes, and response processes. We provide recommended changes to instructions and item wording to improve PHQ-9 validity among Kenyan women.

## Background

Major depressive disorder (MDD) is highly prevalent among women worldwide [1–3]. In sub-Saharan Africa, women are 75% more likely to experience lifetime depression and twice as likely to experience 12-month depression than men [4]. Pregnant and postpartum women have even greater risk of depression, with approximately 35–50% of African women meeting the criteria for MDD during these periods (‘perinatal depression’) [5, 6]. Perinatal depression has been associated with HIV risk behaviors (e.g., unprotected sex, substance use) and adverse health outcomes including HIV acquisition, maternal mortality, and child growth and mortality [7–12].

Depressive symptom screening is a critical first step for identifying women with likely perinatal depression and linking them to care. Screening tools are particularly important in resource-limited settings such as Kenya where it is often not possible to conduct clinical diagnostic interviews (the ‘gold-standard’ for MDD diagnosis) due to shortages in clinician or counselor time, financial resources, and trained mental health personnel. In this context, screening could be integrated in HIV and reproductive health clinics to improve patient access to mental health treatment services [13–15].

A wide variety of depressive symptom screening tools have been developed to provide a basis for a diagnosis of ‘likely’ or ‘probable’ MDD and to triage patients for mental health services [16]. The most commonly used screening tool is the Patient Health Questionnaire-9 item (PHQ-9), which has been previously validated in a wide variety of settings including Kenya and translated into Swahili and numerous other languages. This tool can be administered quickly, accurately measures depression, and directly relates to the nine Diagnostic and Statistical Manual of Mental Health Disorders (DSM-5) criteria for MDD diagnosis [16–21]. The Edinburgh Postnatal Depression Scale (EPDS) has also been widely used among populations of pregnant and postpartum women, with high sensitivity and specificity for detecting major depressive disorder in this population, and may have advantages over the PHQ-9 for detecting perinatal depression because the scale does not include somatic depressive symptoms which could relate to either depression or pregnancy (e.g., changes in appetite or sleeping) [22]. However, while the PHQ-9 has been less frequently used among pregnant and postpartum women [22], several studies have found similar sensitivity and specificity estimates for the PHQ-9 and the EPDS suggesting that women are able to disentangle pregnancy symptoms from somatic depressive symptoms [22–25]. Moreover, because PHQ-9 is often used in the general population, depression screening with the PHQ-9 can facilitate comparisons between pregnant and postpartum women and other groups of women. Finally, the PHQ-9 was recently recommended for widespread use in primary care, reproductive health, and HIV clinics by the Kenyan Ministry of Health as a first step in depressive symptom screening and linkage to care [16].

Despite the PHQ-9’s widespread use in Kenya and elsewhere, prior work has shown that it may not perform well when self-administered or when conducted among women with lower education levels or those who did not learn English as a first language [16, 26–28]. Qualitative research is needed to explore whether PHQ-9 items are understood as intended and culturally relevant and to potentially facilitate the incorporation of cultural idioms of depression for Kenyan women [16]. Cognitive interviewing is a specific type of qualitative method that can be used during questionnaire development, refinement, and translation to ensure that the survey items and their response options capture intended information, to identify potential sources of response error, and to understand how questions are comprehended by survey respondents [29]. This present study sought to conduct cognitive interviews to: 1) qualitatively explore conceptualizations of depression and factors affecting depressive symptoms among a sample of pregnant and postpartum women, half of whom were living with HIV; 2) understand PHQ-9 item acceptability and comprehension; and 3) translate findings into a local adaptation of the PHQ-9 for depression screening among Kenyan women. This work is critical to inform future Kenyan Ministry of Health activities to integrate the PHQ-9 and depression screening and referral services within HIV and reproductive healthcare settings in Kenya, will lend important insights around how participants conceptualize depression and respond to depression screening tool items, and will lead to recommendations on PHQ-9 Kiswahili and Kikuyu translations to potentially improve PHQ-9 sensitivity and specificity among pregnant and postpartum women in this setting.

## Methods

### Participant recruitment

Women were recruited from August 2017 – March 2018 at an HIV prevention and care clinic in Thika, Kenya. They were participants in a larger trial, the Safer Conception Intervention for Partners (SCIP) study (clinicaltrials.gov #NCT03030768), which included 74 mutually-disclosed HIV serodiscordant couples with immediate fertility intentions [30]. SCIP was a pilot intervention study of a comprehensive safer conception package, including integrated antiretroviral therapy (ART), pre-exposure prophylaxis (PrEP), menstrual cycle tracking, and referral for other safer conception services [30]. The SCIP parent study screened a total of 119 women in HIV serodiscordant couples, of whom 83 were eligible for SCIP study participation and 74 were enrolled [30]. Participants were eligible for the SCIP parent study if they were over 18 years of age and less than 50 years of age, expressed a desire for pregnancy in the next 3 years, were sexually active, were willing to enter the study as a couple with their male partner, and intended to remain a couple for the study duration. They were ineligible if they had any indication of subfertility or infertility or if they were pregnant or breastfeeding in the past 3 months.

Active female SCIP participants were eligible to participate in this ancillary study if they were currently pregnant or recently postpartum (delivered a live baby within the last 6 months) at the time of protocol approval. SCIP participants were not pregnant or recently postpartum at the time of enrollment in this parent study, and recruitment for our ancillary study took approximately 1 year because we needed to allow sufficient time for a sample of SCIP participants to become pregnant and deliver their children. All SCIP participants who did become pregnant or who were recently postpartum were invited to enroll during their routine SCIP study visits by a counselor who confirmed their eligibility and obtained written informed consent in their preferred language. Two SCIP study counselors conducted recruitment and enrollment for this SCIP ancillary study and participants were already familiar with these counselors from their time in the SCIP parent study. Recruitment and enrollment for this ancillary study occurred in a private room after completion of all SCIP parent study procedures at a given clinic visit. We recruited pregnant and postpartum SCIP participants, regardless of their HIV status and pregnancy trimester, and we generally waited to recruit and enroll participants for one study visit after their positive pregnancy test (one to 3 months after their pregnancy test) to ensure that we would not have a large sample of women with early miscarriages. We hypothesized that a sample of approximately 20–30 women would be sufficient to elicit a range of PHQ-9 scores and saturation of qualitative themes. Protocols were approved by ethical review boards at the University of Washington and Kenya Medical Research Institute (KEMRI).

### PHQ-9 translation

Kenyan counselors from the clinic site translated the PHQ-9 from English into Kiswahili and Kikuyu. We based the Kiswahili translation on previous work conducted with Kenyan adults but included regional expressions for the dialect commonly spoken in central Kenya [20]. The Kikuyu version was developed by the study’s Kenyan clinical psychologist (JN) and reviewed for accuracy by two other staff members, all Kenyans and native Kikuyu speakers. Versions were back-translated into English and reviewed by mental health professionals in Kenya and the United States to ensure that the translated items maintained fidelity to the meaning of the English items.

### Data collection

We conducted data collection in two phases in order to utilize a retrospective verbal probing approach for cognitive interviewing [29]. In this technique, participants complete the survey with a trained counselor or clinic provider as they would in a “real-world” delivery setting (Phase 1) and then they complete a cognitive interview where a clinical psychologist probed them on their responses to the survey questions and question comprehension and acceptability (Phase 2). We chose this retrospective probing approach over other cognitive interviewing techniques of simultaneous probing (probing after each survey question) or the think-aloud method (asking participants to describe their response processes while they are actively trying to answer each survey question) in order to create a more realistic experience of PHQ-9 administration and to ensure comparability across participant interviews. Retrospective probing is also recommended in later stages of questionnaire development or when testing more established scales with similar response options for each item [29].

In Phase 1, a study counselor administered the PHQ-9 to consenting participants. We chose to interviewer-administer the PHQ-9 (rather than have participants self-administer the scale) because of previous studies’ recommendations about the validity of interviewer-administration [16], desires to provide participants with real-time support if they become distressed by any of the items, and concerns around participant literacy. The items were read aloud in their preferred language (English, Kiswahili, or Kikuyu) and counselors were instructed to read the items exactly as written. After completing the paper form, counselors summed the individual item scores (range: 0–27) and classified participants as having no depressive symptoms (score < 5), minimal symptoms (score 5–14), or moderate to severe symptoms (score > 14) according to previously validated cut-offs [31]. Counselors also completed debriefing reports summarizing words that participants did not understand, any instances where participants asked them to provide definitions or synonyms for a word, and general comments about the overall PHQ-9 administration experience.

In Phase 2, a Kenyan clinical psychologist conducted cognitive interviews with a purposive sample of 20 participants to elicit themes related to conceptualization of mood and factors related to depressive symptoms, PHQ-9 acceptability, item comprehension, and recommended changes to PHQ-9 item wording. These participants were selected to achieve representation of individuals with different PHQ-9 scores, levels of education, preferred languages of administration, pregnancy and postpartum stages, and HIV status. Twenty interviews were sufficient to achieve saturation. Participants were initially recommended for cognitive interviews by the counselor who administered their PHQ-9. The study coordinator and counselors kept track of participant demographic factors after completion of Phase 1 and then referred individuals for Phase 2 in similar proportions to the overall SCIP parent study sample (e.g., 46% of SCIP parent study participants were women living with HIV and we sought to also interview approximately 40% of women living with HIV) [30]. The counselor brought the completed PHQ-9 and debriefing report to the psychologist, who approached potential participants, explained the purpose of the interviews, and reminded participants that interview participation would not affect their participation in the SCIP study or their clinical care. We chose to have a clinical psychologist conduct the cognitive interviews, rather than the study clinical staff who administered the PHQ-9 in Phase 1, because we wanted scale delivery to closely resemble real-world PHQ-9 assessment with a counselor but felt that a different interviewer would elicit more honest responses than the counselor who had seen participants more frequently. The clinical psychologist was also fluent in English, Kiswahili, and Kikuyu and could probe participants in multiple languages during the interview as needed.

Semi-structured cognitive interview guides included scripted probes related to: question comprehension (understanding of what the question asking); decision processes (motivation to respond accurately, sensitivity to the questions); and response processes (match between the participant’s desired answer and response options) for each PHQ-9 item [29]. We also asked participants to describe their experiences completing the survey, perceptions of what the scale was trying to measure, and recommended changes to the items. Finally, we asked generally about participants’ conceptualization of mood and factors related to depressive symptoms. The clinical psychologist was also instructed to use the PHQ-9 tool and debriefing report information to probe participants on specific items that they had difficulty with or places where they asked the counselor to define a word. We piloted the guide with staff to ensure cultural appropriateness and clarity of questions. The final guide was translated into Kiswahili and Kikuyu.

Interviews were conducted immediately after the PHQ-9 administration to reduce recall issues. In cases when we were unable to conduct the interviews immediately after PHQ-9 data collection, we refreshed participants on the items and their responses prior to the interview. Interviews were conducted in English, Kiswahili, and/or Kikuyu and lasted approximately 45 min. Data collection took place in a quiet, private area and interviewers took detailed notes on debriefing forms. All interviews were audio-recorded, transcribed, and translated into English. Participants who endorsed depressive symptoms were referred to further mental health evaluation and individuals who were deemed to be a harm to themselves or others were immediately linked to medical care with close follow up from study staff. Participants received travel reimbursement for attending their SCIP parent study visits and also received additional travel reimbursement for this ancillary study if their cognitive interview was conducted on a different day than their SCIP clinic visit.

### Data analysis

We used descriptive statistics to summarize the sample. These quantitative analyses were conducted using SAS 9.4 (Cary, North Carolina, USA).

Qualitative analyses focused on understanding key themes around conceptualization of depression and PHQ-9 comprehension, decision processes, and response processes based on the model of Willis and colleagues [29]. We conducted a multi-stage analysis, whereby we first developed participant-level analytic memos summarizing themes from interviews and debriefing reports for each PHQ-9 item [32]. These memos included representative quotations, and we consulted the original Kiswahili and Kikuyu interviews to accurately convey participants’ views. We summarized data from these memos in matrix format to identify issues with each PHQ-9 item by participant demographics (i.e., HIV status, pregnant or postpartum, education, PHQ-9 score, and language of administration) [33]. Issues with the items were assigned to one of three categories: question comprehension; decision processes; or response processes. In addition, we reviewed transcripts and participant-level memos to identify general themes related to scale administration and acceptability (summarized in a separate matrix), and wrote analytic memos on participants’ conceptualization of depressive symptoms and factors related to mood. All analytic memos and matrices were reviewed by the primary analyst (JV), a secondary analyst (NT, RM, or SA), and the study clinical psychologist (JN), and we sought broader feedback on our results from HIV and reproductive health care providers in Kenya. Discrepancies in findings were resolved by discussion. Based on these analyses and team discussions, we drafted revised versions of the PHQ-9 for future testing among pregnant and postpartum Kenyan women.

## Results

### Participant characteristics

We approached 30 women and enrolled 29 (97%) for PHQ-9 administration. Of these, 16 (55%) were postpartum and 13 (45%) were living with HIV (Table 1). The median level of education was 8 years (IQR 8, 12). Most participants preferred to have the PHQ-9 administered in Kiswahili (*N* = 15; 52%). Approximately 11 women (38%) had PHQ-9 scores < 5, 17 (59%) had scores between 5 and 14, and 1 (4%) had a score > 14. The most commonly endorsed PHQ-9 item asked about “trouble falling asleep, staying asleep, or sleeping too much” (*N* = 21; 72%). A total of 4 participants (14%) endorsed the item regarding suicidal ideation in the prior 2 weeks and all of them were immediately linked to medical care and followed closely by study personnel. Approximately 79% of participants living with HIV had PHQ-9 scores ≥5 (median PHQ-9 score of 6), compared with only 50% of HIV-uninfected participants (median PHQ-9 score of 4). PHQ-9 scores were similar between pregnant and postpartum women (61% of pregnant women had PHQ-9 scores ≥5 compared with 62% of postpartum women).

**Table 1:** Participant characteristics for participation in PHQ-9 completion and cognitive interview

Characteristic	Frequency	
	Phase 1 PHQ-9 Completion (*N* = 29)	Phase 2, Cognitive Interview (*N* = 20)
Age, years	29.7 (26.4–33.7)	29.3 (25.2–33.3)
Any income reported	19 (65.5%)	12 (60.0%)
Education, years	8.0 (8.0–12.0)	10.5 (8.0–12.0)
Literate	29 (100.0%)	20 (100.0%)
Preferred language of PHQ-9		
English	14 (48.3%)	12 (60.0%)
Kiswahili	15 (51.7%)	8 (40.0%)
Kikuyu	0 (0.0%)	0 (0.0%)
Preferred language of cognitive interview		
English	NA	0 (0.0%)
Kiswahili		6 (30.0%)
Kikuyu		2 (10.0%)
Mix of two or more languages		12 (60.0%)
Married or in a relationship	27 (93.1%)	20 (100.0%)
Partnership duration, years^a^	1.6 (0.8–6.2)	1.3 (0.5–5.6)
Number of prior children	1 (1–2)	1 (0–2)
Any unprotected sex with current partner in prior month	7 (24.1%)	5 (25.0%)
Any sex with outside partner in prior month	1 (3.5%)	0 (0.0%)
HIV-infected	13 (44.8%)	8 (40.0%)
On ART, participants living with HIV only	13 (100.0%)	8 (100.0%)
On PrEP, HIV-uninfected participants only	14 (87.5%)	12 (100.0%)
Pregnancy status		
1st Trimester	6 (20.7%)	3 (15.0%)
2nd Trimester	5 (17.2%)	3 (15.0%)
3rd Trimester	2 (6.9%)	2 (10.0%)
Postpartum	16 (55.2%)	12 (60.0%)
Frequency of individual PHQ-9 items^b^		
Little interest or pleasure	17 (58.6%)	12 (60.0%)
Feeling down, depressed, hopeless	16 (55.2%)	11 (55.0%)
Trouble sleeping too little or too much	21 (72.4%)	14 (70.0%)
Tired or little energy	15 (51.7%)	11 (55.0%)
Poor appetite or overeating	18 (62.1%)	14 (70.0%)
Feeling bad about yourself	7 (24.1%)	6 (30.0%)
Trouble concentrating	9 (31.0%)	7 (35.0%)
Moving slowly or feeling restless	7 (24.1%)	6 (30.0%)
Thoughts of suicide of self-harm	4 (13.8%)	2 (10.0%)
Median PHQ-9 score	5 (3–9)	5 (3–9.5)
PHQ-9 scoring categories		
< 5	11 (37.9%)	8 (40.0%)
5–14	17 (58.6%)	11 (55.0%)
> 14	1 (3.5%)	1 (5.0%)

Data are number (%) or median (IQR). *PHQ-9* Patient Health Questionnaire 9-item, *ART* antiretroviral therapy, *PrEP* pre-exposure prophylaxis

^a^Partnership duration was assessed by asking all participants when they first had sex with their study partner and calculating the time between first sex with partner and the enrollment date

^b^Endorsement of each PHQ-9 item reflects symptom experiences in the prior 2 weeks

From the sample of 29 women, we purposively selected 20 (69%) to complete cognitive interviews, all of whom agreed to participate. These participants had a median of 10.5 years of education (IQR 8, 12), and preferred to conduct the interview in Kiswahili (*N* = 6; 30%) or a mix of two or more languages (*N* = 12; 60%). The majority were postpartum (*N* = 12; 60%) and 8 (40%) were living with HIV. A total of 8 (40%) participants had PHQ-9 scores < 5, 11 (55%) had PHQ-9 scores between 5 and 14, and 1 (5%) had a PHQ-9 score > 14.

### Conceptualization of depressive symptoms and factors related to mood

Five themes emerged related to conceptualization of depressive symptoms and factors related to mood (Table 2). “Depression” was typically well understood and when participants were asked to explain depression in their own words they often used cultural idioms including “thinking too much”, “feeling moodless”, “feeling like your head will burst”, and “having a lot of disturbing thoughts.” Depressive symptoms were also thought to be quite transient during pregnancy (“tomorrow you will find that your mind is stable and you are back to normal”) and were related to external stressors including responsibilities as a wife and a mother, relationship with a partner, and one’s HIV status. For example, several participants described feeling depressed when they failed to take care of house chores, when they were concerned with food security or their child’s education, or when they were fighting with their partners. One participant described an experience when she had suicide intent and “bought poison” after she fought with her partner because she worried that “he will leave me at some point”. Participants living with HIV described times when they experienced depressive symptoms because they were worried about transmitting HIV to their child (“you keep thinking about your child’s results”), experienced HIV-related stigma (“society stigmatizes [HIV-infected people] so they get depression”), or thought their partner would leave them because of their HIV status (“I think about my husband leaving me because he probably sees me differently”). When specifically talking about feeling like a failure or letting one’s family down, several participants discussed their family’s disappointment when they acquired HIV or learned they were pregnant and had to drop out of school. Finally, those who experienced depression often talked about the importance of prayer and religious coping for improving mood (“you tell God that He’s in control…and you begin to feel peace”). Religious convictions were also discussed as a barrier to harming oneself (“let Him take you when the time comes instead of doing yourself harm”).

**Table 2:** Summary of key themes related to conceptualization of mood and factors related to depressive symptoms

Key theme	Representative quotations
“Depression” is described with specific cultural idioms including “thinking too much”, “feeling moodless”, and “feeling like your head will burst”	“You feel like your head is going to burst, you are thinking about a lot of issues. You are feeling so depressed.” *Pregnant, HIV-uninfected, spoke in Kiswahili and English, PHQ-9 score of 8* “Depression is when you are having a lot of disturbing thoughts.” *Postpartum, HIV-infected, spoke in Kiswahili and English, had a PHQ-9 score of 2*
Depressive symptoms are common but transient during pregnancy and postpartum periods	“If you find that for two days you don’t feel the same way you were a few days ago, you get a counselor to talk to you because we women, we feel so moodless when we are pregnant and it’s not intentional. Sometimes you will find your husband asking for food and you tell him to go and get for himself the food and this is just because you are moodless, it’s not because you want to. But tomorrow you will find that your mind is stable and you are back to normal.” *Pregnant, HIV-uninfected, spoke in Kiswahili and English, PHQ-9 score of 8*
Depressive symptoms are related to external stressors including responsibilities as a wife and mother, relationship with a partner, and one’s HIV status	“Sometimes one feels they are down because you want to do that thing but you don’t make it. Like you can see I want to wash dishes but I don’t feel like it so I will feel like I am down. Yes, the house is dirty, what the husband will think, you see?” *Pregnant, HIV-uninfected, spoke in Kikuyu, Kiswahili, and English, PHQ-9 score of 4* “Sometimes you find that you are quarrelling with your husband. You find that the children have been chased away from school for school fees, no food is in your house. You feel you are so stressed…if it’s eating, there is no mood to eat. When people talk to you, you feel that today you have no mood.” *Pregnant, HIV-uninfected, spoke in Kiswahili and English, PHQ-9 score of 8* “I might wake up and sit with my husband, we might start chatting and then you find we have disagreed over a small issue, or he asks me to do something and then I tell him that I will not be able to do it but he insists. That issue affects me and I see as if he is forcing me to do something that I don’t want to do. I feel bad about it…I keep getting depressed because of these issues. Sometimes I lose hope as to whether we will stay together or if he will leave me at some point. There was a time when we had a disagreement and I found myself going to get poison to take. Luckily he came home and so I did not take it. I find myself thinking about it [still].” *Pregnant, HIV-infected, spoke in Kiswahili and English, PHQ-9 score of 9* “Most of the time, the way I am (HIV-positive), you keep thinking about your child’s results…My child has already been tested for [HIV] and I had a lot in my mind about the results. Something else that can bother my mind is when you live discordant, I think about my husband leaving me because he probably sees me differently.” *Postpartum, HIV-infected, spoke in Kiswahili and English, PHQ-9 score of 5* “Mostly what can make a person die is not using [HIV] medication wrongly, it is lack of counseling and stigma from HIV negative people because they can be using medicine well but those around them stigmatize them or society stigmatizes them so they get depression or stress. It is mainly about feeling like a failure especially when you look at your age mates, when you compare your life and see that their lives are better.” *Postpartum, HIV-infected, spoke in Kiswahili, PHQ-9 score of 17*
Unintended pregnancy and HIV diagnosis can cause women to feel they’ve let their families down	“I can use an example, whereby we have a family, they had a girl child, so the girl child fails to finish her education, she gets pregnant. For me that can be a failure. Disappointing the family.” *Postpartum, HIV-uninfected, spoke in Kiswahili and English, PHQ-9 score of 3* “Mostly it’s about being HIV positive so it makes me think I have let people down, so I feel like even if I do something good, I doubt that anyone will see as if I have achieved anything.” *Postpartum, HIV-infected, spoke in Kiswahili, PHQ-9 score of 17*
Religious coping is important for participants who experience depressive symptoms, feelings of hopelessness, or suicidal ideation	“[Depression] is when you say that you have lost hope. What can I say, like I had said earlier, you should just thank God…When I experience the things I have told you and I am not able to eat, I ask God to help me. If I face any difficulty, God will help me.” *Pregnant, HIV-uninfected, spoke in Kikuyu, PHQ-9 score of 8* “With depression it’s something that has really gotten to you, that’s weighing heavily on the mind and you feel that thing might bring harm to your body. However, after some days you can get into a state of acceptance, you begin to see that God is there, you pray, you tell God that He’s in control, that He’s the one that can intervene and you begin to feel peace.” *Pregnant, HIV-uninfected, spoke in Kiswahili, PHQ-9 score of 8* “[When talking to someone with suicidal intent], I would comfort them and tell them because it was God who created you let Him take you when the time comes, instead of doing yourself harm which could mean that you are correcting God”. *Pregnant, HIV-infected, spoke in Kiswahili and Kikuyu, PHQ-9 score of 2*

### PHQ-9 comprehension, decision processes, and response processes

Almost all interview participants reported that the PHQ-9 was acceptable and “easy to complete” because they felt that “it is measuring those things that most women experience”. Several (*N* = 4) said that the PHQ-9 could serve as an intervention for depressive symptoms because the questions helped to normalize mental health issues and made them feel that someone was interested in their lives:

*“The questions make me feel at peace. They are a source of comfort. It makes me feel that I am not alone in this because when I am given this question, I know there are others who also have [depression] and that is comforting. You (the interviewer) are also a source of comfort.”* (Pregnant, HIV-infected woman with a PHQ-9 score of 2)

When asked whether they would prefer the PHQ-9 as a self- or interviewer-administered questionnaire, most participants said that they would rather it be interviewer-administered because they enjoyed talking with the interviewer and wanted to be able to ask clarifying questions (“when alone I may fail to understand some parts, but if we are two we can discuss about it”) (*N* = 19). We did not detect differences in PHQ-9 comprehension, decision, or response processes by pregnancy or postpartum stage, HIV status, or education level of the participants.

#### Item comprehension

Participants were able to understand the content and wording of most items, in both the English and Kiswahili versions, but had some difficulties distinguishing between concepts asked in different questions and relating the concepts to their own lives (Table 3). For item 1 (“little interest or pleasure in doing things”), participants discussed tasks that they felt it was their duty to complete but that they typically do not enjoy (e.g., “house chores”, “work”, forced “sex” with a partner, “taking care of children”) (*N* = 9). Their lack of enjoyment of these tasks was often reported as a constant in their lives, rather than being related to recent mood issues, and participants commonly described endorsing this item even if their experience of little interest or pleasure was not limited only to the past 2 weeks. This lends context to the quantitative data which showed that item 1 was one of the most commonly endorsed PHQ-9 items. Similarly, some participants interpreted item 4 (“tired or little energy”) as having little energy specifically to do house chores and found this item to be repetitive with question 1.

**Table 3:** Summary of findings by PHQ-9 item and types of cognitive process problems discussed (*N* = 20)

Item (N women who had comprehension, decision process, or response process problems with a given item)	Comprehension problems: What does the participant think the question is asking?	Decision process problems: Does the participant want to tell the truth and/or devote mental energy to the question?	Response process problems: Can the participant map her internal answer with a given answer choice?^a^
1. Little interest or pleasure in doing things (*N* = 9)	Often related it to tasks that women feel they should do but don’t enjoy (e.g., household chores, work)	Reluctance to endorse this item if it meant failing to perform “duties”	None
2. Feeling down, depressed, hopeless (*N* = 2)	None	Some hesitation to endorse if participants felt they should “turn to God for acceptance” when experiencing these feelings	None
3. Trouble falling asleep, staying asleep, or sleeping too much (*N* = 5)	None	None	Difficulty responding for those who experienced only one type of sleep issue, or if it occurred for part of a day (e.g., during a nap)
4. Tired or little energy (*N* = 3)	Comprehended similarly to item 1 and participants discussed feeling little energy to do household chores or work	Reluctance to endorse this item if it meant failing to perform “duties”	None
5. Poor appetite or overeating (*N* = 4)	None	None	Participants were generally not sure how to respond if they experienced only one type of appetite issue, or if it occurred for part of a day (e.g., during a midday meal)
6. Feeling bad about yourself—or that you are a failure or have let your family down (*N* = 5)	None	Reluctance to endorse this item if it meant failing to perform “duties”	None
7. Trouble concentrating on things such as reading the newspaper or watching television (*N* = 8)	Comprehension difficulties among participants who could not relate to given examples	None	Difficulty responding for those who experienced this issue for only part of a day, or if they did not own a television or read the newspaper regularly
8. Moving or speaking so slowly that other people could have noticed, or the opposite—being so fidgety or restless that you have been moving around more than usual (*N* = 17)^b^	Difficulty understanding the word “fidgety” on the English PHQ-9 (a direct translation of this word was not available for the Kiswahili PHQ-9 translation).	None	Difficulty responding for those who experienced only one type of issue, or if it occurred for part of a day
9. Thoughts that you would be better off dead or of hurting yourself in some way (*N* = 2)	None	Some hesitation to endorse if participants felt they should “turn to God for acceptance” when experiencing these feelings	None

^a^Response processes problems were most often mentioned by participants who endorsed a given item (and generally those who had higher PHQ-9 scores) as these individuals experienced a symptom but then may have experienced difficulty in identifying an appropriate response

^b^Comprehension and response process problems differed meaningfully by language of administration. Specifically, participants who received the English PHQ-9 had difficulty understanding the word “fidgety” but this comprehension issue was mentioned less frequently among participants who completed the Kiswahili PHQ-9

For question 7 (“trouble concentrating on things such as reading the newspaper or watching television”), participants had difficulties relating “trouble concentrating” to the examples provided, particularly if they did not own a television or regularly read the newspaper, and they more often talked about trouble concentrating as it related to their ability to “complete housework” or “attend church” (*N* = 8). Finally, among those who completed the English PHQ-9, the word “fidgety” was almost universally not understood (*N* = 17). All other items were well comprehended and participants were able to describe the concepts in their own words (often switching between English, Kiswahili, and Kikuyu) during the cognitive interview. Participants generally understood the purpose of the questionnaire and were able to disentangle pregnancy and HIV symptoms from depressive symptoms (e.g., “I am hungry all the time but it is because of this pregnancy”).

#### Decision processes

Participants reported social desirability bias as a reason for difficulty in accurately answering five of the PHQ-9 questions (Table 3). Specifically, several participants were hesitant to endorse items 1 (*N* = 9), 4 (*N* = 3), and 6 (*N* = 5) because it would be an admission to both the interviewer and themselves that they had failed to complete their “duties” as a wife and mother. While most participants found items 2 (“feeling down, depressed, or hopeless”) and 9 (“thoughts that you would be better off dead”) acceptable and culturally appropriate, a few participants who described themselves as “church going” said that they would not endorse those items because “even if I feel those feelings I know I should turn to God for acceptance” (*N* = 4).

#### Response processes

Response process problems were the most commonly reported issues. Participants had difficulty responding to the double-barreled questions in items 3 (“trouble falling asleep, staying asleep, or sleeping too much”; *N* = 5), 5 (“poor appetite or overeating”; *N* = 4), and 8 (“moving or speaking so slowly…or the opposite”; *N* = 5) if they had only experienced one type of symptom mentioned in the question or if they experienced that issue for only part of a day (e.g., difficulty sleeping during a nap but not at night; Table 3). In addition, participants were hesitant to choose a response to item 7 (“trouble concentrating”) if they experienced difficulty concentrating in other areas of their lives besides watching television or reading (e.g., difficulty interacting with friends in peer groups or “chamas”, completing chores, praying) (*N* = 8). When asked about their overall feelings on the response options, participants who experienced several symptoms and who had higher PHQ-9 scores (PHQ-9 score > 5) reported difficulties choosing between “several days” and “more than half the days” and keeping all four of the response options in mind while answering a question. Participants also had challenges identifying a two-week period (some identified a shorter time period while others considered the whole prior month) and choosing a response option when a problem did not occur on consecutive days during that period.

#### Suggested changes to the PHQ-9

**Fig. 1:**
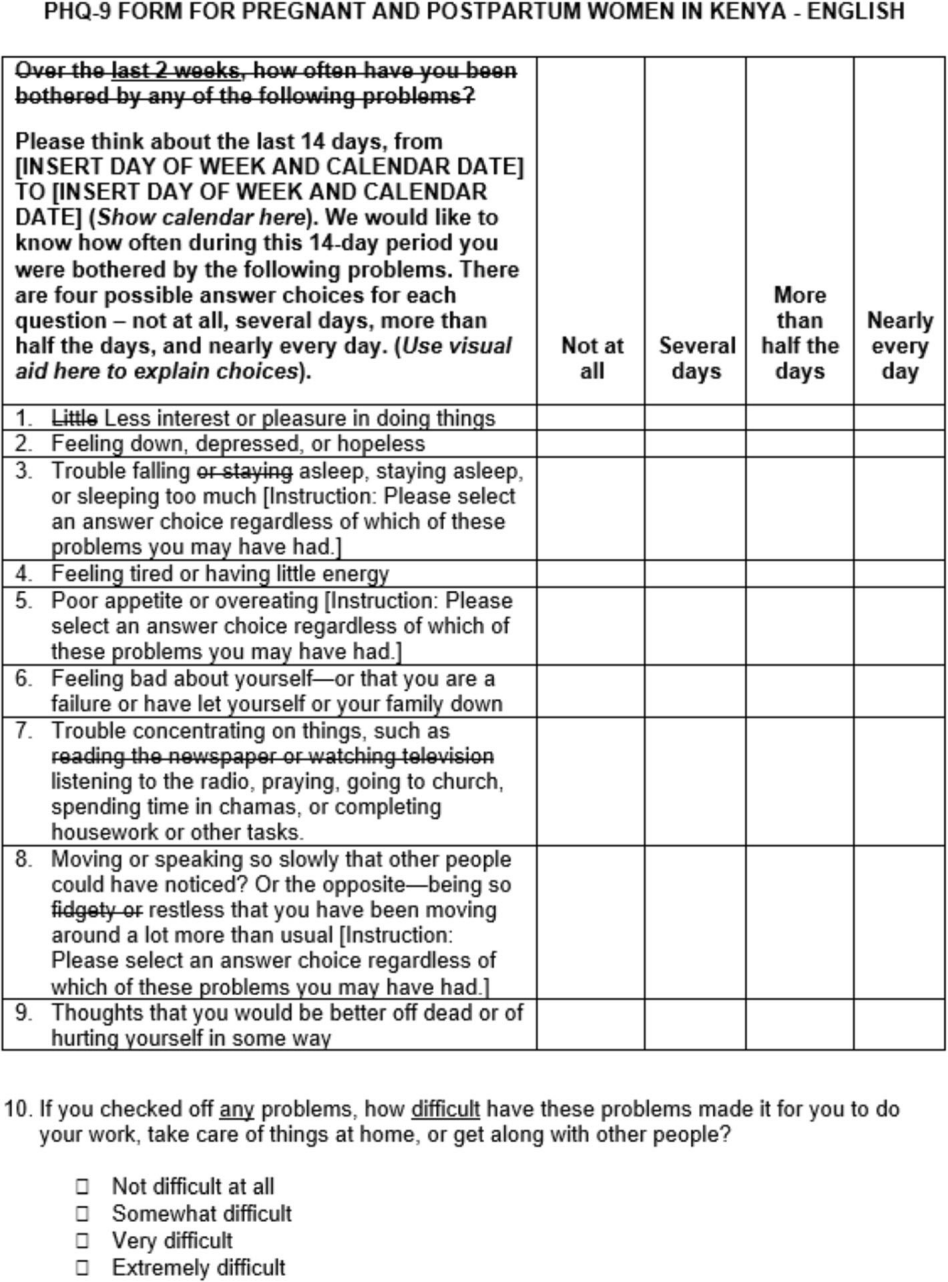
Suggested English PHQ-9 revisions

Based on our qualitative findings and team discussions, we made several changes to the English and Kiswahili PHQ-9 tools that will potentially mitigate issues around comprehension, decision processes, and response processes while still preserving the meaning and content of the validated scale items (Fig. 1). First, changing the word “little” to “less” in item 1 may help participants distinguish between tasks that they typically do not enjoy doing and those that they have recently been less interested in. Second, additional instructions for items 3, 6, and 8 may help participants select an answer choice regardless of whether they experienced only one of the symptoms included in the double-barreled questions. Third, more culturally relevant examples for item 7 are important for allowing participants to identify with the question and choose an appropriate response. Finally, we propose removing the word “fidgety” from the English version of the PHQ-9 item 8 when it is delivered to comparable non-native English-speaking patient populations.

**Fig. 2:**
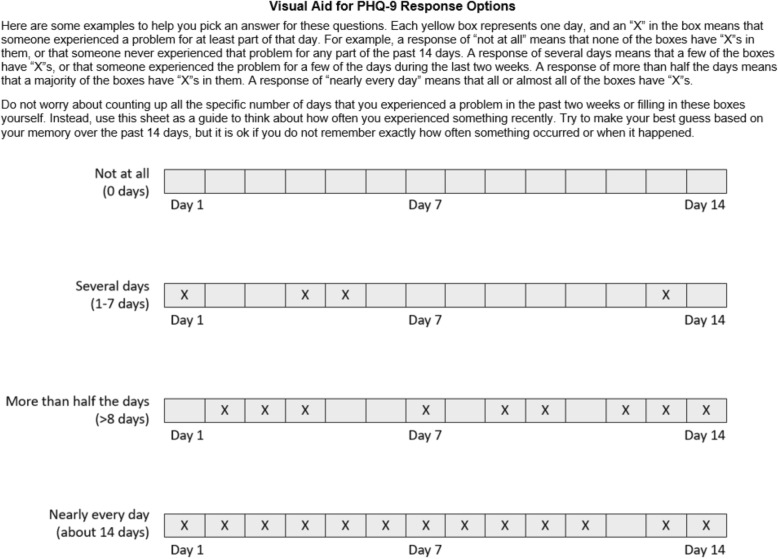
Suggested instructional tool to aid participants in selecting a PHQ-9 response option

We have also developed additional instructional language for describing the two-week reference time period and response options (Fig. 1) and a visual aid to help participants understand the difference between the response options (Fig. 2) in settings where the PHQ-9 is to be delivered as an interviewer-administered tool.

## Discussion

In this study among pregnant and postpartum women in Kenya, some of whom were living with HIV, the PHQ-9 was acceptable as an interviewer-administered screening tool. Some items lacked full comprehension and we recommend key changes for these items and additional instructions for double-barreled questions. Participants also reported difficulty choosing between several response options and we have developed a visual aid for use in similar settings where the PHQ-9 is interviewer-administered. These results provide encouraging findings on the broader use of the PHQ-9 for depression screening among pregnant and postpartum women in Kenya, lend insights to participant thought processes while they respond to PHQ-9 items, and highlight opportunities to improve scale validity as its use is being currently scaled-up by the Kenyan Ministry of Health.

Previous qualitative studies of PHQ-9 comprehension and decision and response processes have similarly reported that patients may have difficulty identifying appropriate responses, particularly when they have experienced a symptom for at least some time in the prior 2 weeks, and the PHQ-9 response choices may not accurately capture their symptom severity [34, 35]. Patients have described difficulty selecting responses when their symptoms fluctuated prior to the clinic visit and they also experienced some social desirability and recall bias in selecting response options [35]. These findings may help to explain why PHQ-9 scores in this sample were lower than expected and few women had scores > 14 (the cut-off typically used to identify moderate to severe depressive symptoms), despite describing significant depressive symptoms and suicide ideation during the interviews [16]. Other studies of the PHQ-9 in Asia and Africa have identified a PHQ-9 score of 10 as the optimal cutoff for identifying patients with MDD in resource-limited settings [36–38], and we would have classified an additional five women with moderate to severe depressive symptoms using this cutoff. Future research is necessary to improve both item and instruction wording and guidance around clinically relevant cut-off scores in Kenya.

Previous studies of depression screening have debated whether to include the sensitive and potentially stigmatizing item related to suicidal ideation, despite known associations between depressive symptoms and suicide [39, 40]. While we found some evidence of social desirability bias related to this item, participants did not object to inclusion of the question or find it uncomfortable to answer. In addition, participants who endorsed this item accurately described times when they had thoughts of committing suicide, indicating that the item was well comprehended despite previous findings about its misinterpretation [41]. Women in our cohort reported hesitancy to endorse feelings of suicidal ideation particularly if they were religious, commensurate with other studies in East Africa that have described the influence of religious beliefs on suicidal ideation and behavior [42, 43]. Religious adults in Uganda and Ghana have described suicide as being “unacceptable” and against God’s rule, but still sympathized with and tried to help individuals in their community with suicidal ideation [38, 39]. However, healthcare staff administering the PHQ-9 may need additional training on asking about suicidal ideation in a nonjudgmental manner, identifying referral locations for mental healthcare, and linking individuals to immediate care [44, 45].

PHQ-9 items related to somatic depressive symptoms (e.g. issues with appetite or sleeping) may be difficult to distinguish from symptoms of HIV or pregnancy [28, 39]. We did not detect a difference in median PHQ-9 scores or our cognitive interview findings by HIV status or pregnancy or postpartum stage (although the frequency of participants with PHQ-9 scores ≥5 differed by HIV status), and pregnant women were often able to distinguish somatic symptoms related to their pregnancy (e.g., nausea in the first trimester, trouble sleeping with a newborn baby) from those related to depression. Other research has shown that there may be little benefit to removing somatic items and these items are likely especially important in identifying depressive symptoms among East African women who experience or talk about somatic depressive symptoms more often than men and women in Western settings [27, 39, 46–50].

The strengths of this study included the two-stage data collection from a well-established cohort of pregnant and postpartum women who were already comfortable talking with study staff. We also had strong referral systems to link patients with a mental healthcare provider as needed and no adverse events were reported during the study. Limitations included our small sample size of individuals with immediate fertility intentions who were in stable, mutually-disclosed HIV serodiscordant relationships. It was not always possible to conduct cognitive interviews immediately after questionnaire administration and the time lag between PHQ-9 completion and interviews may have impacted some participant responses. We only had one individual with a PHQ-9 > 14, which may have limited our ability to understand differences in depression experiences and PHQ-9 understanding by symptom severity. Also, our results may not be generalizable to a population of women newly diagnosed with HIV or those who have unintended pregnancies. We did not conduct follow-up cognitive interviews with the revised PHQ-9 or compare PHQ-9 scores with the EPDS, also commonly used for perinatal depression screening. However, recent studies have found that the EPDS performs similarly to the PHQ-9 in pregnant and postpartum populations [23–25]. Future large-scale validation studies comparing the revised PHQ-9 to clinical diagnostic interviews for MDD diagnosis among pregnant and postpartum women in East Africa are necessary. It will also be critical to conduct additional mixed methods research to assess acceptability and comprehension for the our revised PHQ-9 and corresponding instructional tool, with both target participants and healthcare providers who would be administering the PHQ-9 under Kenyan Ministry of Health guidelines. Anthropological studies of individual experiences, feelings, and thoughts labeled as depressive symptoms, and social, religious, and cultural experiences around these symptoms, may help to identify cultural mediators of depression in this setting. Finally, there remains a need to conduct provider training and provide support on the best approaches to administer PHQ-9 items in a culturally appropriate and understanding manner and ensure that appropriate referral services are available and accessible to patients.

## Conclusions

The PHQ-9 is an acceptable and well-comprehended screening tool for perinatal depression among HIV-infected and uninfected women in Kenya. However, we detected several shortcomings in its comprehension and the associated processes for decision and response. In light of this, we provide recommendations for PHQ-9 item wording, response option instructions, and a participant-facing visual aid. This work highlights the importance of using cognitive interviewing methods to explore participants’ understanding of depressive symptom screening tools and conceptualization of factors related to mood. Future research is needed to test our revised PHQ-9 and patient-facing instructional tool with both patients and healthcare providers in Kenya and this is a critical next step for our work. While our findings are context-specific, our methods are generalizable to other settings and populations and cognitive interviewing may be a particularly useful approach to refine the PHQ-9 for low-literacy populations and individuals who do not speak English as a first language [16]. Further refinement of the PHQ-9 tool and integration of depression screening into HIV and reproductive healthcare has the potential to improve maternal and child health outcomes.

## Data Availability

The datasets used and/or analyzed during the current study and the translated Kiswahili version of the PHQ-9 questionnaire are available from the corresponding author on reasonable request.

## Abbreviations

ART: Antiretroviral therapy
DSM-5: Diagnostic and Statistical Manual of Mental Health Disorders, 5th Edition
EPDS: Edinburgh Postnatal Depression Scale
KEMRI: Kenya Medical Research Institute
MDD: Major Depressive Disorder
PHQ-9: Patient Health Questionnaire-9 item
PrEP: Pre-exposure prophylaxis
SCIP: Safer Conception Intervention for Partners
